# Intraoperative Ketorolac and Outcomes after Ovarian Cancer Surgery

**DOI:** 10.3390/jcm13061546

**Published:** 2024-03-07

**Authors:** Mathieu Luyckx, Céline Verougstraete, Mathieu Jouret, Kiswendsida Sawadogo, Marc Waterkeyn, Frédéric Grandjean, Jean-Paul Van Gossum, Nathanael Dubois, Vincent Malvaux, Lucie Verreth, Pascale Grandjean, Pascale Jadoul, Charlotte Maillard, Amandine Gerday, Audrey Dieu, Patrice Forget, Jean-François Baurain, Jean-Luc Squifflet

**Affiliations:** 1 UNGO (UCLouvain Network of Gynaecological Oncology), 1200 Brussel, Belgium; marcwaterkeyn@yahoo.fr (M.W.); f.grandjean@europehospitals.be (F.G.); jpvangossum@clstjean.be (J.-P.V.G.); ndubois@clstjean.be (N.D.); vincent.malvaux@cspo.be (V.M.); lucie.verreth@cspo.be (L.V.); grandjeanvw.pascale@gmail.com (P.G.); jean-francois.baurain@saintluc.uclouvain.be (J.-F.B.); jean-luc.squifflet@saintluc.uclouvain.be (J.-L.S.); 2 Gynaecological Oncology Board, Institut Roi Albert II, Cliniques Universitaires Saint-Luc, UCLouvain, 1200 Brussels, Belgium; celine.verougstraete@student.uclouvain.be (C.V.); pascale.jadoul@saintluc.uclouvain.be (P.J.); charlotte.maillard@saintluc.uclouvain.be (C.M.); amandine.gerday@saintluc.uclouvain.be (A.G.); 3 TILS Group, De Duve Institute, UCLouvain, 1200 Brussels, Belgium; 4 Obstetrics and Gynecology Department, Centre Hospitaliser de Wallonie Picard (CHWaPi), 7500 Tournai, Belgium; mathieu.jouret@chwapi.be; 5 Statistical Support Unit, Cliniques Universitaire Saint-Luc, UCLouvain, 1200 Brussels, Belgium; kiswendsida.sawadogo@saintluc.uclouvain.be; 6 Obstetrics and Gynecology Department, Cliniques de l’Europe, St. Elisabeth Branch, 1180 Brussel, Belgium; 7 Obstetrics and Gynecology Department, Cliniques de l’Europe, St. Michel Branch, 1040 Brussel, Belgium; 8 Obstetrics and Gynecology Department, Clinique St-Jean, 1000 Brussel, Belgium; 9 Obstetrics and Gynecology Department, Clinique St-Pierre, 1340 Ottignies, Belgium; 10 Obstetrics and Gynecology Department, Centre Hospitalier Régional, 7000 Mons, Belgium; 11 Anesthesiology Department, Cliniques Universitaire Saint-Luc, UCLouvain, 1200 Brussels, Belgium; audrey.dieu@saintluc.uclouvain.be; 12 Epidemiology Group, Department of Anaesthesia, School of Medicine, Medical Sciences and Nutrition, Institute of Applied Health Sciences, University of Aberdeen, NHS Grampian, Aberdeen AB24 3UE, UK; 13 Medical Oncology Department, Institut Roi Albert II, Cliniques Universitaires Saint-Luc, UCLouvain, 1200 Brussels, Belgium

**Keywords:** ovarian cancer, oncological surgery, peri operative inflammation, NSAID

## Abstract

Introduction: Surgery is the cornerstone of ovarian cancer treatment. However, surgery and perioperative inflammation have been described as potentially pro-metastagenic. In various animal models and other human cancers, intraoperative administration of non-steroidal anti-inflammatory drugs (NSAIDs) appears to have a positive impact on patient outcomes. Materials and methods: In this unicentric retrospective study, we provide an exploratory analysis of the safety and potential benefit of intraoperative administration of ketorolac on the outcome of patients undergoing surgery for ovarian cancer. The study population included all patients who were given a diagnosis of ovarian, fallopian tube or peritoneal cancer by the multidisciplinary oncology committee (MOC) of the Cliniques universitaires Saint-Luc between 2015 and 2020. Results: We included 166 patients in our analyses, with a median follow-up of 21.8 months. Both progression-free survival and overall survival were superior in patients who received an intraoperative injection of ketorolac (34.4 months of progression-free survival in the ketorolac group versus 21.5 months in the non-ketorolac group (*p* = 0.002), and median overall survival was not reached in either group but there was significantly higher survival in the ketorolac group (*p* = 0.004)). We also performed subgroup analyses to minimise bias due to imbalance between groups on factors that could influence patient survival, and the group of patients receiving ketorolac systematically showed a better outcome. Uni- and multivariate analyses confirmed that administration of ketorolac intraoperatively was associated with better progression-free survival (HR = 0.47 on univariate analysis and 0.43 on multivariate analysis, *p* = 0.003 and 0.023, respectively). In terms of complications, there were no differences between the two groups, either intraoperatively or postoperatively. Conclusion: Our study has shown a favourable association between the use of ketorolac during surgery and the postoperative progression of ovarian cancer in a group of 166 patients, without any rise in intra- or postoperative complications. These encouraging results point to the need for a prospective study to confirm the benefit of intraoperative administration of ketorolac in ovarian cancer surgery.

## 1. Introduction

Ovarian cancer (OC) is estimated to be the seventh most common cancer in women and the eighth most common cause of cancer death, with five-year survival rates below 45% [1,2]. The risk of developing ovarian cancer increases with age and peaks around 65 years old [2]. The high mortality rate of ovarian cancer is due to features like the asymptomatic and secret growth of the tumour, the delayed onset of symptoms, and often the lack of proper screening, resulting in its diagnosis at an advanced stage [3].

Several risk or protective factors for epithelial ovarian cancer have been identified, most of them relating to reproductive and hormonal factors like parity, family history or genetic factors (BRCA1/BRCA2), use of oral contraception, endometriosis and smoking [2,3,4].

Nowadays, the standard approach to treat ovarian cancer includes aggressive surgery with complete cytoreduction and chemotherapy with doublet carboplatin and paclitaxel [5,6]. Still, the proportion of women dying from ovarian cancer has moderately improved over time [2]. Therefore, new treatments with modified strategies are actively being searched for [5]. The last decade has seen new molecules validated in OC with improvement in the control of the disease, such as anti-angiogenic agents [7] or poly (ADP ribose) polymerase (PARP) inhibitors, the second of which has been associated, as shown in the SOLO 1-study, with an improvement in the progression-free survival of patients with BRCA mutations and advanced ovarian cancer [8]. Furthermore, PARP inhibitors have also been tested in women with Homologue Recombination Deficiency (HRD) in the PRIMA trial studying niraparib, and the results also showed a statistically significant improvement in the PFS [9].

Ovarian cancer, like many other epithelial cancers, spreads by direct extension to adjacent organs and disseminates throughout the peritoneal cavity. The peritoneal spread may be quite extensive and thus, the surgical removal of peritoneal implants can lead to significant tissue disruption and inflammation.

Because complete cytoreductive surgery is often a major surgery, with multi-organ resection and extended peritonectomies, several studies have concentrated on the impact of induced perioperative inflammation on the evolution of the disease. It has been suggested that such cytoreductive surgeries, as well as the associated inflammatory reaction, can facilitate the metastatic process [5,10,11].

Surgery modifies the tumour environment in ways that may promote tumour cell dissemination, survival and expansion [5]. Increasing evidence suggests that surgery can promote metastasis, not simply by mechanical dissemination of cancer cells but also by stimulation of systemic inflammation and surgery-associated immunosuppression, resulting in outgrowth of dormant cancer cells at distant sites [12,13,14,15].

Several mechanistic hypotheses have been advanced. The manipulation of the tumour and its environment will result in the liberation of pro-inflammatory substances like prostaglandins (PGE2), thromboxane and cytokines [16]. This PGE2 production has been shown to have an influence on T cell proliferation, T cell activation and also the development of myeloid-derived suppressive cells (MDSCs) [17]. In ovarian cancer, the role of MDSCs appears to be very central for the control of the disease by the immune system [18]. Surgery may also have another impact on cell-mediated immunity through tumour-associated neutrophils that generate reactive oxygen species, prostaglandins and vascular endothelial growth factor (VEGF) [14].

### 1.1. Anti-Inflammatory Drugs in Ovarian Cancer (OC)

The important impact of inflammation in the development of cancer and metastasis has led to the evaluation of the value of administering anti-inflammatory treatment around the time of diagnosis. Several studies have shown that the use of aspirin and other non-steroidal anti-inflammatory drugs (NSAIDs) reduces the development of carcinomatosis and the risk of death from ovarian cancer [19,20]. A recent cohort study demonstrated that users of aspirin or NSAIDs after the diagnosis of OC induced better cancer-specific survival compared to never users [21]. In colorectal cancer, aspirin use has also been proven to reduce specific cancer mortality [22].

### 1.2. Ketorolac and Ovarian Cancer

Ketorolac was approved by the US Food and Drug Administration (FDA) in 1989 as the first injectable NSAID [23]. Unlike most NSAIDs, ketorolac contains a mix of the two enantiomers of a molecule (S-enantiomer and R-enantiomer) in equal proportions, with the S-forms having inhibitory activity toward cyclo-oxygenase (COX) 1 and 2 [24]. By inhibiting COX enzymes, intraoperative ketorolac administration may reduce inflammation and its downstream mediators known to be associated with the initiation and progression of the disease [25], and it may also reduce aberrant expression of COX in OC [26] and its negative consequences and might then be a good strategy to improve its treatment.

Years ago, in our institution, routine perioperative ketorolac administration was implemented in cancer surgery. We were very interested in investigating whether the administration of a single intraoperative dose of ketorolac during a debulking surgery for ovarian, tubal or peritoneal cancer was safe and could be associated with longer progression-free survival (PFS) and overall survival (OS).

## 2. Materials and Methods

This retrospective cohort study was coordinated by the Department of Gynaecology of the Cliniques universitaires Saint-Luc (CUSL), Brussels, Belgium, and patients were recruited within the UNGO (UCLouvain Network of Gynaecological Oncology) [27]. Ethical approval for this study (Ethical Committee N/REF 2018/21FEV/067) was provided by the IRB (CEBH of the Université catholique de Louvain, Brussels, Belgium. Chairperson Pr J.M. Maloteaux) on 5 March 2018.

The study population included all patients who were given a diagnosis of ovarian, fallopian tube or peritoneal cancer by the multidisciplinary oncology committee (MOC) of the Cliniques universitaires Saint-Luc between 2015 and 2020.

Inclusion criteria in the study comprised surgery (cytoreduction for advanced cases or restaging surgery for early-stage disease) with postoperative anatomo-pathologic confirmation of the diagnosis and the possibility of having a follow-up period.

Patients who did not undergo surgery at all or who did not undergo surgery in our institution were excluded. Other exclusion criteria encompassed immediate death after surgery, patients who did not receive chemotherapy despite it being justified because of their general condition, and non-epithelial histology.

The selected patients were divided into two groups depending on whether they had a perioperative administration of ketorolac or not. The administration of ketorolac was left to the discretion of the anaesthesist in charge and the protocol they chose preoperatively. All the patients who underwent laparotomy surgery received epidural thoracic anaesthesia (except 21 patients, due to technical reasons or contra-indication).

For both groups, a large number of variables, quantitative and qualitative, were extracted from their medical records and compiled in a database. Those variables included, among others, some factors related to prognosis (e.g., age, Ca-125, Sugarbaker, neo-adjuvant chemotherapy, residual disease and FIGO stage), type of treatment, administration of morphine drugs pre- and postoperatively, postoperative complications and oncological evolution.

PFS and OS were calculated from the date of diagnosis, whether the treatment started with chemotherapy or surgery.

### Statistical Methods

Patients’ baseline characteristics are presented as median [IQR] for continuous variables that were not normally distributed or as number [n, percentage] for discrete variables. Survival lengths are presented as median [IQR]. Categorical variables were compared between groups using the Chi Square test or the Fisher exact test and continuous ones with the Mann–Whitney test. A univariate Cox model and log-rank test were used to assess the potential impact of these baseline characteristics and to investigate a potential association with ketorolac use. Kaplan–Meier analyses were used to estimate PFS and OS probabilities. After univariate analysis, a multivariate Cox regression model was created, adjusting for any relevant baseline, intraoperative and oncological factors related to the outcome. We used backward stepwise regression and all factors significant at a *p*-value ≤ 0.05 were retained in the final model with a higher *p*-value bound for inclusion in the multivariate model of 0.20. STATISTICA (data analysis software system) version 7 (Statsoft Inc. 2004, Tulsa, OK, USA) and SAS 9.4 were used for all analyses.

## 3. Results

We analysed a total of 166 patients in our study. The median follow-up of the cohort was 21.8 (13.5–32.5) months. The characteristics of the patients are detailed in Table 1.

In the ketorolac group, patients were statistically younger (59, range 51–70) compared to the non-ketorolac group (71, range 62–76), with a significant difference (*p* < 0.001). The ketorolac group also showed statistically fewer residual diseases at the end of surgery (5.4% versus 16.7%, *p* = 0.039), including 11% supra centimetric residue (CC2–CC3) in the non-ketorolac group compared to 0.9% in the ketorolac group. Additionally, fewer patients in the ketorolac group received neoadjuvant chemotherapy (46.4% versus 63%, *p* = 0.046) but none received Hyperthermic Intraperitoneal Chemotherapy (HIPEC), whereas 5.6% in the non-ketorolac group benefited from HIPEC. No differences were observed in FIGO stage, histological type, CA125 level, disease grade, BRCA mutation status, initial volume of ascites and maintenance therapy (PARPi or Bevacizumab) received after chemotherapy.

Only 3% of patients with epidural thoracic anaesthesia also received Patient Controlled Analgesia (PCA) with opioids. During surgery, 75% of patients in both groups received opioids. In the postoperative periods, 79% of the patients in the non-ketorolac group received opioids and 83% in the ketorolac group, which does not represent a statistically significant difference between groups for opioid use.

**Figure 1:**
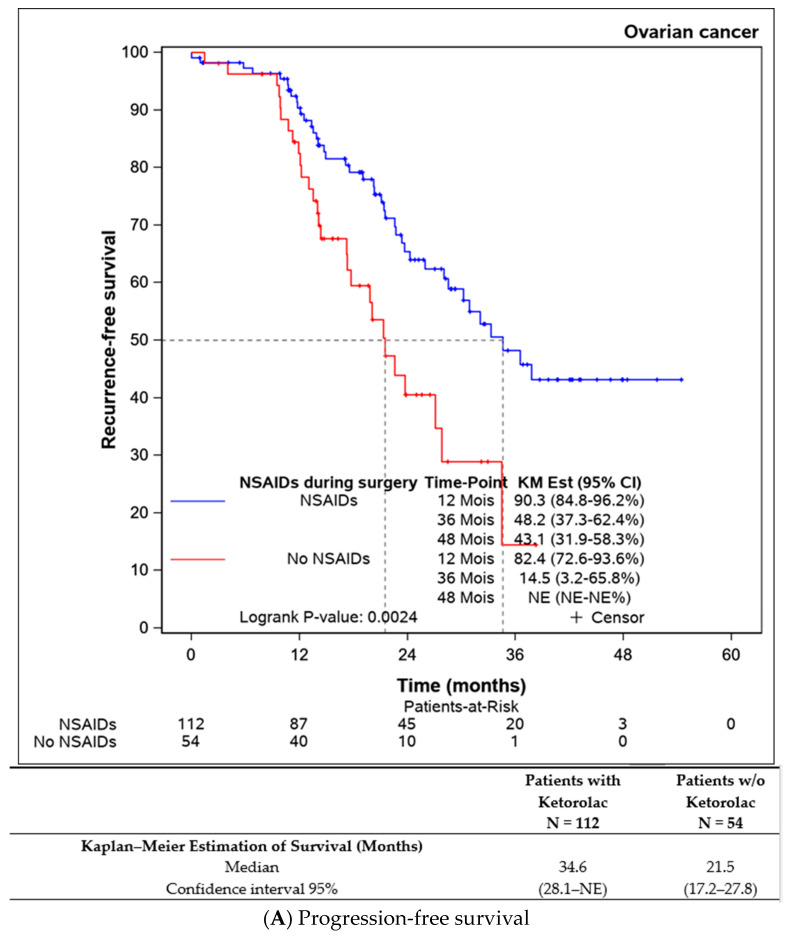

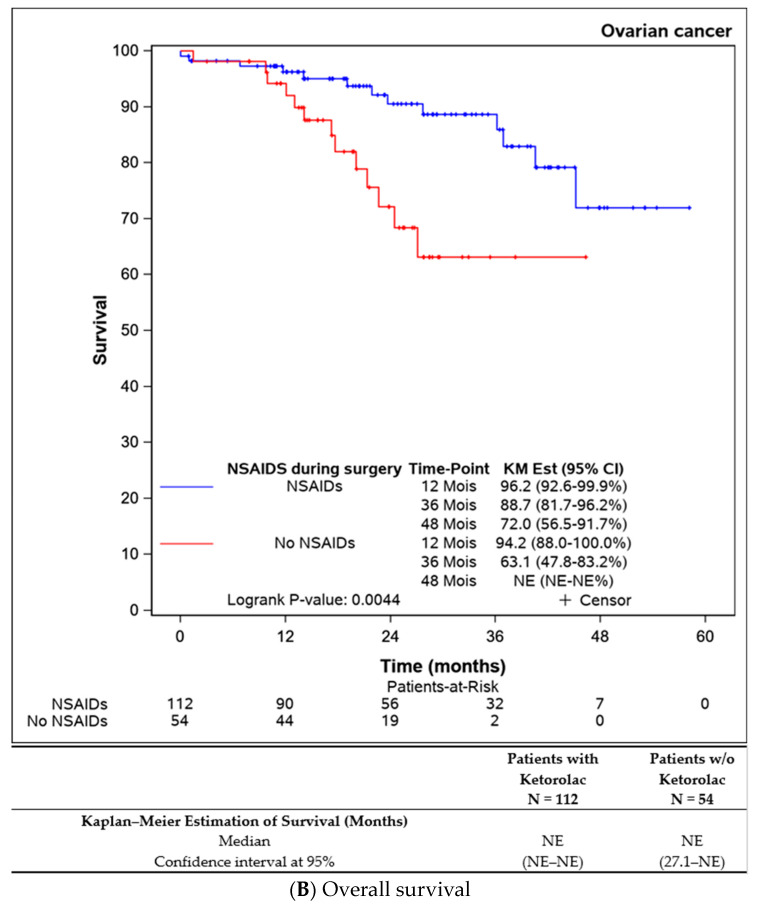
Comparison of the progression-free survival and overall survival between the patients receiving ketorolac during the surgery or not.

Both progression-free survival and overall survival were superior in patients who received an intraoperative injection of ketorolac (Figure 1A,B). The ketorolac group exhibited a median progression-free survival of 34.6 months (28.1-NE), compared to 21.5 months (17.2–27.8) in the non-ketorolac group (*p* = 0.002). Median overall survival was not reached in either group, but the ketorolac group demonstrated a statistically significant higher survival (*p* = 0.004).

**Figure 2:**
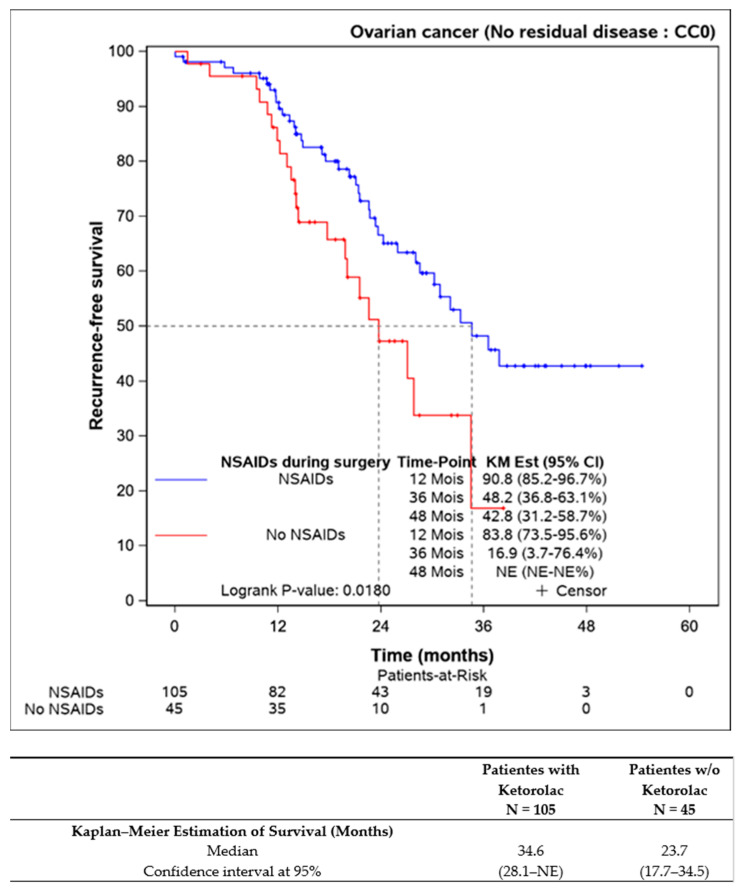
Progression-free survival in completely resected patients, according to the ketorolac administration or lack thereof (n = 150).

Survival comparisons were also conducted on patients who underwent complete resection of the disease (n = 150), revealing statistically improved progression-free survival for those receiving perioperative ketorolac (34.6 (28.1–NE) months) versus non-ketorolac (23.7 (17.7–34.5] months, *p* = 0.018) (Figure 2).

**Figure 3:**
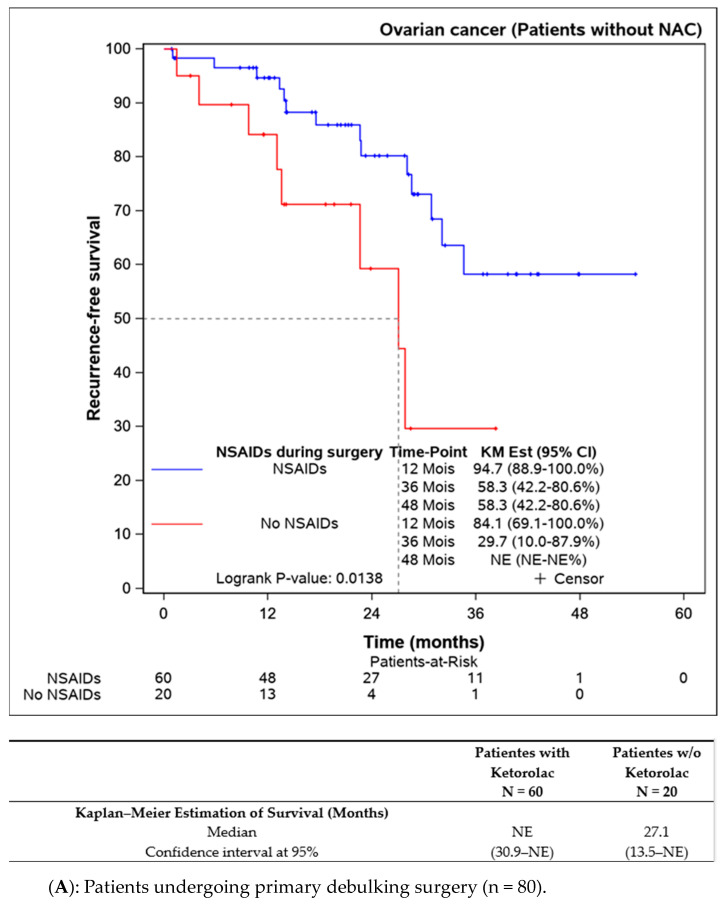

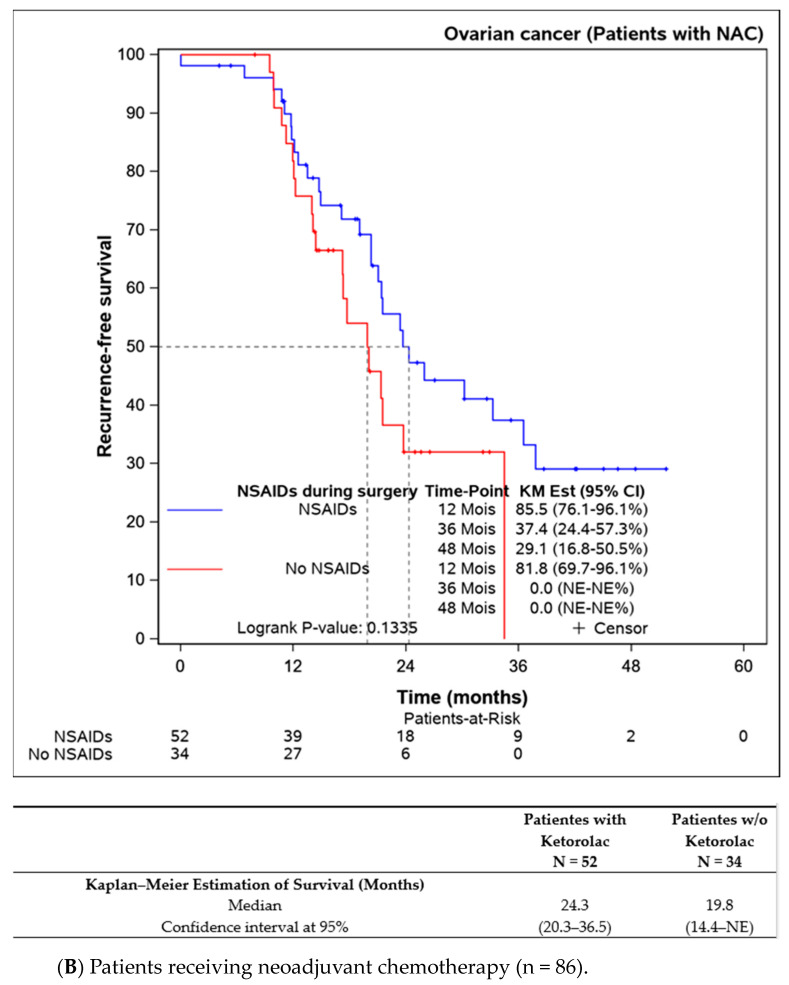
Progression-free survival in patients undergoing primary debulking surgery (without neoadjuvant chemotherapy) according to the ketorolac administration (**A**) and patients receiving neoadjuvant chemotherapy before surgery (**B**).

Subgroup analyses were performed on patients not receiving neoadjuvant chemotherapy (n = 109), indicating better progression-free survival in the ketorolac group (median not reached) versus the non-ketorolac group (27.1 (13.5–NE) months, *p* = 0.014). Among patients undergoing neoadjuvant chemotherapy, the difference between groups was not significant, though progression-free survival appeared to be better in the ketorolac group (24.3 months versus 19.3 months, *p* = 0.134) (Figure 3A,B).

**Figure 4:**
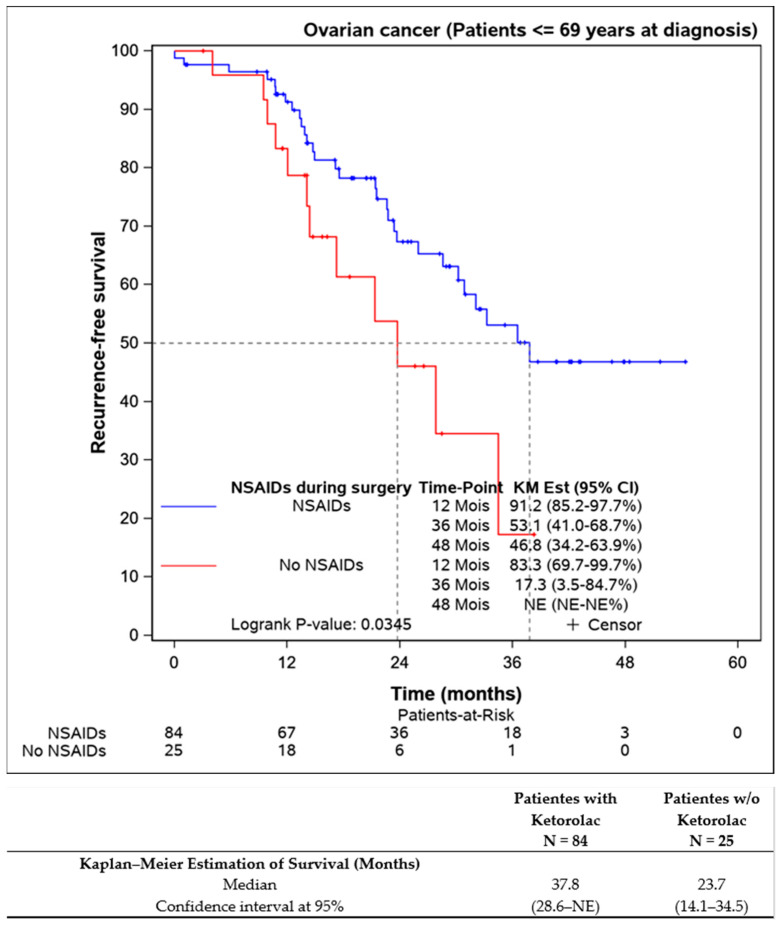
Progression-free survival of the patients younger the 69 years old according to the Ketorolac administration. (n = 109).

Further analysis of patients younger than 69 years aimed to eliminate age bias, confirming that ketorolac administration was associated with better progression-free survival (37.8 (28.6–NE) months) compared to the non-ketorolac group (23.7 (14.1–34.5) months) (Figure 4).

**Figure 5:**
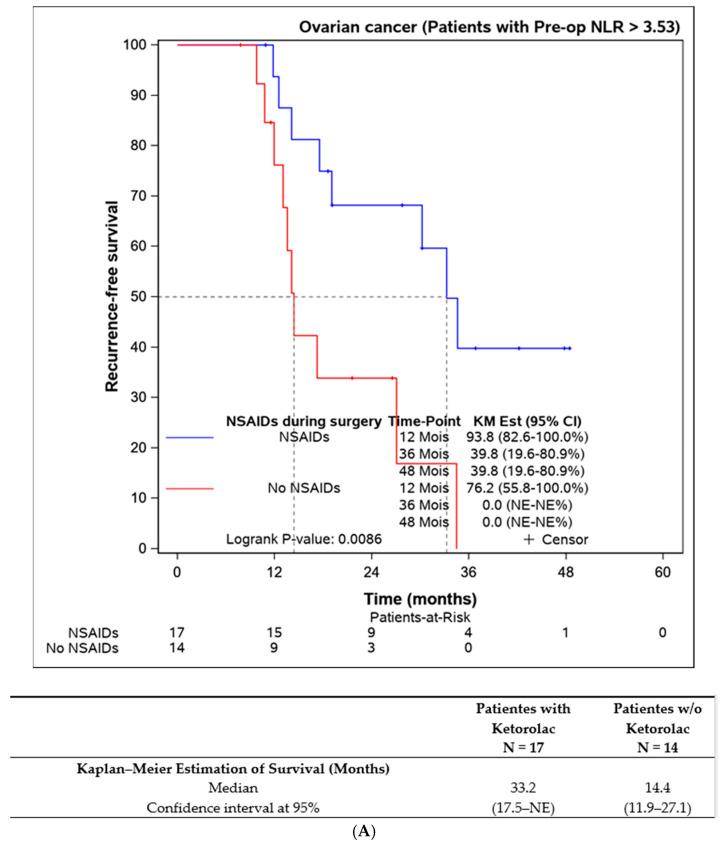

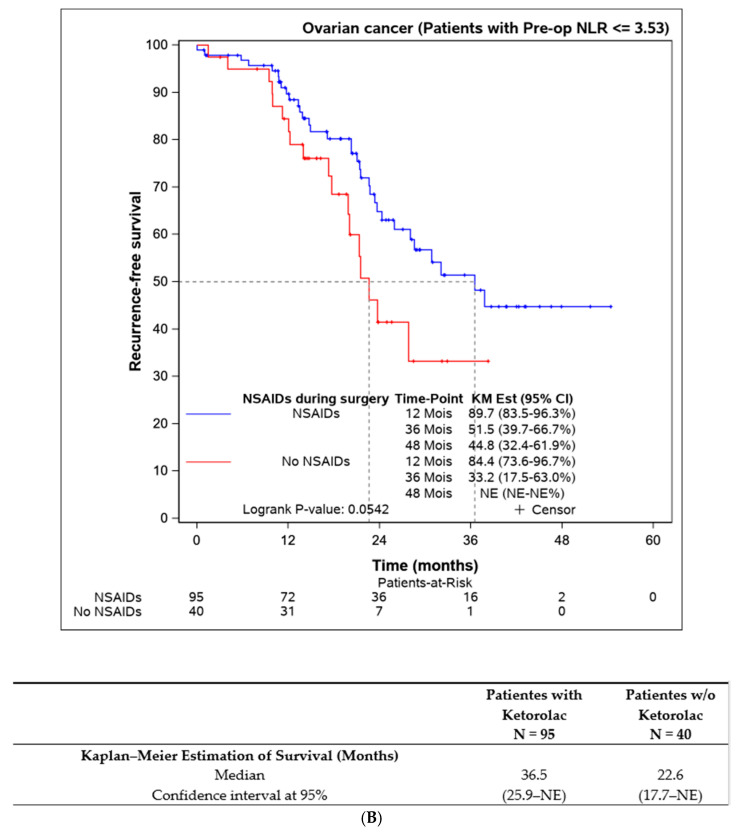
Progression-free survival according to the preoperative NLR and the influence of ketorolac administration during the surgery. (**A**) Disease-free survival in patients with NLR > 3,53 according to the administration of ketorolac (n = 31). (**B**) Progression-free survival in patients with NLR ≤ 3,53 according to the administration of ketorolac (n = 135).

Lastly, we assessed the efficacy of perioperative ketorolac in patients with elevated inflammation (neutrophil-to-lymphocyte ratio > 3.53) at the time of surgery. A statistically significant difference in progression-free survival was found in favour of the ketorolac group (33.2 (17.5–NE) months) compared to the non-ketorolac group (14.4 (11.9–27.1) months, *p* = 0.009). This difference was not observed in patients with an NLR < 3.53 (Figure 5).

We performed uni- and multivariate analyses (Table 2) and, in both analyses, administration of ketorolac was associated with an improvement in progression-free survival (univariate analysis Hazard Ratio = 0.47, *p* = 0.003; multivariate analysis Hazard Ratio = 0.43, *p* = 0.023).

Regarding complications during and after surgery, no significant differences were observed between the two patient groups (Table 3, Table 4 and Table 5).

## 4. Discussion

We present a retrospective cohort study indicating that the administration of ketorolac during cytoreductive surgery for ovarian cancer is associated with an increase in both progression-free survival (PFS) and overall survival (OS). Given the retrospective nature of the study and the absence of randomisation, the two groups were not well balanced for several factors known to impact survival in ovarian cancer patients. The control group had a higher average age, more residual disease at the end of surgery and a greater incidence of neoadjuvant chemotherapy.

Subgroup analyses were subsequently conducted to address the imbalance in factors potentially influencing progression-free survival attributable to ketorolac administration. With the exception of patients who received neoadjuvant chemotherapy, where the difference was not statistically significant, we confirmed that patients who received ketorolac during their procedure demonstrated improved disease-free survival, thus mitigating potential biases associated with age, residual disease, and neoadjuvant chemotherapy. Uni- and multivariate analyses also confirmed that the administration of ketorolac was statistically significantly associated with an improvement in progression-free survival.

We also explored the impact of patients’ initial general inflammation prior to surgery on the efficacy of ketorolac administration in the perioperative period, and we were able to confirm that the higher the level of pre-existing inflammation, the greater the outcome improvement after ketorolac administration. NLR has already been published as a performant marker of inflammation and a predictor of responses to ketorolac administration [28].

Surgery induces an inflammatory reaction, modifying the tumour environment in ways that may promote tumour cell dissemination, survival and expansion [5,29]. The manipulation of the tumour and its environment will result in the liberation of pro-inflammatory substances like prostaglandins (PGE2), thromboxane and cytokines [16]. Prostaglandins, as well as thromboxane, are lipid signalling molecules that can promote cell proliferation, angiogenesis, and metastasis and inhibit apoptosis [5]. PGE2 also inhibits antigen presentation and suppresses NK cells, overall inhibiting anti-tumour immunity and hence promoting tumour escape from dormancy [12,30,31]. It has been shown that cyclo-oxygenase (COX) enzymes, which transform arachidonic acid in prostaglandins, are overexpressed in some ovarian cancers [5,14].

Cytokines such as IL1-beta and IL-8 contribute to an increase in the release of vascular adhesion molecules and the production of VEGF and other growth factors, therefore promoting angiogenesis and metastasis [14,32].

Finally, it is known that natural killer cells (NK cells), which have the spontaneous capacity to eliminate tumour cells and are thus involved in the resistance to metastasis, are very vulnerable during surgery [14,33].

Recent studies suggest that non-steroidal anti-inflammatory drugs (NSAIDs) could impact inflammation during and after surgery, and consequently, the related immune dysfunction [34,35].

A potential mechanism could be the inhibition of COX enzymes by the NSAIDs, which will result in reduced prostaglandin levels.

There are two isoforms of COX enzymes known as COX-1 and COX-2. They seem to operate in a coordinated manner in cancer pathophysiology, especially in the tumorigenesis process. COX-2 is generally considered to be the inducible isoform, responsible for enhanced prostanoid production in response to inflammatory stimuli and growth factors during inflammation and various pathological conditions, including cancer. COX-1 has been suggested to be the major enzyme regulating PGE_2_ production in ovarian cancer cells [36,37,38,39].

Ketorolac, which is a simple and inexpensive NSAID, has been shown to preferentially inhibit COX-1. It exhibits a lower COX-2 activity [12,40,41]. As already mentioned in the introduction, ketorolac contains a mix of the two enantiomers of the molecule (S-enantiomer and R-enantiomer) in equal proportions, with the S-forms having inhibitory activity toward cyclo-oxygenase (COX) 2. The R-enantiomers of ketorolac have nearly no action on COX but have been shown to inhibit Rac1 and Cdc42 [42]. They are members of the Ras-homologous (Rho) family of small GTPases and play key roles in cell organisation, cell interactions with the extracellular matrix, adhesion and invasion [43], and central function in the development of cancerous lesions. RAC1 overexpression in OC has been shown to be associated with worse prognosis [44], and it is overexpressed in high-grade serous OC in comparison with low-grade OC [45].

The R-enantiomer of ketorolac was found in high concentration in the peritoneal fluid [45], at a concentration sufficient to inhibit RAC1 and Cdc42. In a mouse model, ketorolac administration was found to prolong mouse survival [5], and in a surgically stressed mouse model implanted with primary syngeneic Lewis lung carcinoma (LLC), ketorolac administration has been shown to reduce the development of dormant micro-metastases and prolong survival of the mouse. This action has been shown to be through COX-1 inhibition (and persistent COX-2 activity) and restoring anti-tumour activity [12]. It has to be noted that in mice, the S-enantiomer of ketorolac is converted to R-ketorolac, which does not occur in humans [46], but as both enantiomers have shown an interesting anti-cancer effect, ketorolac remains a very good candidate to look at.

In the bone marrow, PGE2 induces the differentiation of myeloid precursors into myeloid-derived suppressor cells (MDSCs) that can inhibit T cell activation and stimulate regulatory T cells’ expansion and activity [17]. Again, Panigrahy et al. [12] showed, in their mouse model of metastatic lung cancer, that a single preoperative, but not postoperative, dose of ketorolac administered in mice suppressed lung micrometastases present at the time of primary tumour resection, as described in the previous paragraph. They also noted synergistic anti-tumour activity of ketorolac and resolvins without over-toxicity. They suggested in their paper that the anti-tumour activity of ketorolac is in part driven by COX-1/thromboxane A2 (TXA2) inhibition and the subsequent reduction in platelet aggregation and degranulation. However, they also established the importance of maintaining COX-2 activity to control tumour recurrence or metastasis. The inhibition of COX-2 by celecoxib (which is a selective COX-2 inhibitor) hindered the anti-tumour activity of ketorolac via impairment of the resolution of inflammation [12].

The moderate benefit observed with the administration of ketorolac in patients who have undergone neoadjuvant chemotherapy can be attributed to several hypotheses. Firstly, a high initial level of inflammation is recognised as a factor that diminishes the response to neoadjuvant chemotherapy and adversely affects the overall survival of the patient [47,48]. Secondly, platinum-based chemotherapy and paclitaxel are recognised for activating multiple inflammation pathways, including NFkB, TNFa and Toll-like receptor-4. These pathways contribute to a diminished response to chemotherapy and play a role in the development of chemoresistance in cancer cells [49]. Importantly, these pathways differ from those on which ketorolac is known to exert its activity. Thirdly, the reduction in tumour volume and the disappearance of ascites following neoadjuvant chemotherapy in responsive patients, even to a moderate extent, lead to a reduction in the extent of surgery and the inflammatory environment that contributes to ascites production. In cases of very extensive disease requiring extensive peritonectomy and organ resection, there is significant tissue disruption, a known factor that enhances the metastatic process [50].

In humans, Hudson et al. showed, in a trial involving 123 ovarian cancer patients of whom 14% received perioperative ketorolac, a difference in mortality at a 60-month follow-up (18% of the ketorolac vs. 43% of the non-treated patients, *p* = 0.09) [5]. Retrospective analyses of tumour recurrence in patients undergoing breast cancer surgery have revealed that preoperative administration of ketorolac was associated with a marked reduction of recurrence and mortality after surgery [51]. Of note is that ketorolac did not exhibit cancer-preventive activity when administered postoperatively, which is when NSAIDs are routinely administered for pain management [51]. Guo et al. (2015) also suggested that ketorolac may provide a survival benefit to ovarian cancer patients through inhibition of COX enzymes [6]. Intriguingly, a recent randomised placebo-controlled trial did not confirm any effect of a single dose of ketorolac administered during breast cancer surgery. A possible explanation, even if speculative, may be linked to the inflammatory profile of the tumours in patients at lower risk than expected [52].

### Strengths and Weakness

This retrospective study presents real-life data, demonstrating the safety of administering ketorolac during cytoreductive surgery for ovarian cancer and suggesting potential survival benefits for our patients. Additionally, we offer subgroup analyses in which ketorolac was consistently associated with improved outcomes. These promising exploratory findings motivate the initiation of a prospective validation trial, leveraging these results to identify the optimal patient groups for inclusion and confirm the enhanced survival outcomes associated with intraoperative administration of ketorolac. Our study has methodological limitations, especially its retrospective design and thus a possible selection bias. The follow-up period may have been too short for the patients included in 2020. Finally, the NSAIDs were administered depending on the preference of the anaesthesiologist in charge of the patient, without randomisation, creating unbalanced groups for known factors impacting the survival of patients with ovarian cancer.

Further studies are needed to confirm the positive association between the administration of ketorolac and PFS and OS. Ideally, such studies should use a prospective design to address some of the limitations mentioned above.

## 5. Conclusions

Our study has shown a favourable association between the use of ketorolac during surgery and the postoperative progression of ovarian cancer in a group of 166 patients, without any rise in intra- or postoperative complications. These results are consistent with the observation that many of the pathways promoting tumour growth are activated by the inflammation concomitant to the surgery.

## Figures and Tables

**Table 1:** Patients’ characteristics.

	Patients with AINS N = 112	Patients w/o AINS N = 54	*p*-Value
Age—Median (P25-P75)			<0.001 *
Years	59 (51–70)	71 (62–76)	
**FIGO stage at the diagnosis—no. (%)**			0.493
I	14 (12.5%)	4 (7.4%)	
II	14 (12.5%)	4 (7.4%)	
III	56 (50.0%)	31 (57.4%)	
IV	26 (23.2%)	15 (27.8%)	
Missing	2 (1.8%)	0 (0.0%)	
**Histology—no (%)**			0.710
Serous/serous papillary	82 (73.2%)	43 (79.6%)	
Mucinous	4 (3.6%)	3 (5.6%)	
Endometriod	11 (9.8%)	3 (5.6%)	
Clear cells	2 (1.8%)	0 (0.0%)	
Undifferentiated	2 (1.8%)	0 (0.0%)	
Other	4 (3.6%)	3 (5.6%)	
Missing	7 (6.3%)	2 (3.7%)	
**Neoadjuvant chemotherapy—no. (%)**			0.046 *
Yes	52 (46.4%)	34 (63.0%)	
No	60 (53.6%)	20 (37.0%)	
**Residual disease after surgery—no. (%)**			0.039 *
Yes	6 (5.4%)	9 (16.7%)	
No	105 (93.8%)	45 (83.3%)	
Missing	1 (0.9%)	0 (0.0%)	
**Amount of residual disease (CC classification)—no (%)**			0.005 *
CC0-CC1	110 (98.2%)	48 (88.9%)	
CC2-CC3	1 (0.9%)	6 (11.1%)	
Missing	1 (0.9%)	0 (0.0%)	
**BRCA mutation—no (%)**			0.322
No mutation	30 (26.8%)	11 (20.4%)	
BRCA 1 or BRCA 2 mutation	8 (7.1%)	6 (11.1%)	
Missing	74 (66.1%)	37 (68.5%)	
**Initial CA-125—Median (P25-P75)**			0.203
CA-125 (UI/L)	285 (68–711)	366 (142–1442)	
**Tumour grade—no (%)**			0.077
Low grade (I-II)	18 (16.1%)	3 (5.6%)	
High grade (III)	82 (73.2%)	46 (85.2%)	
Missing	12 (10.7%)	5 (9.3%)	
**Initial ascites—no. (%)**			0.440
Yes	61 (54.5%)	37 (68.5%)	
No	23 (20.5%)	10 (18.5%)	
Missing	28 (25.0%)	7 (13.0%)	
**Inital ascites volume—no. (%)**			0.943
No ascites	23 (20.5%)	10 (18.5%)	
0–500 ml	32 (28.6%)	21 (38.9%)	
500 mL—1 litre	7 (6.3%)	4 (7.4%)	
1–3 litres	8 (7.1%)	4 (7.4%)	
>3 litres	14 (12.5%)	8 (14.8%)	
Missing	28 (25.0%)	7 (13.0%)	
**HIPEC—no. (%)**			0.028 *
Yes	0 (0.0%)	3 (5.6%)	
**No**	32 (28.6%)	12 (22.2%)	
**Missing**	80 (71.4%)	39 (72.2%)	
Maintenance therapy			0.999
Olaparib o Niraparib	12 (10.7%)	5 (9.3%)	
Bevacizumab	2 (1.8%)	1 (1.9%)	
**Median intiale PCI (Sugarbaker)—Median (P25-P75)**			0.177
PCI (0–39)	9.0 (3.0–16.0)	13.0 (5.5–19.0)	
**Median PCI (Sugarbaker) at the debulking—Median (P25-P75)**			0.464
PCI (0–39)	9.0 (3.0–16.0)	9.0 (5.0–17.0)	

* indicates a significant *p*-value at the 5% threshold. Tests used: For continuous variables = Wilcoxon or Mann or Whitney test; For discrete variables = Chi-square test or Fisher test when minimum expected number < 5; HIPEC = Hyperthermic Intraperitoneal Chemotherapy. PCI = peritoneal carcinomatosis index.

**Table 2:** Uni- and multivariate regression analyses on progression-free survival.

		Univariate			Multivariate	
Factor	*p*-Value	Hazard Ratio	HR 95CI	*p*-Value	Hazard Ratio	HR 95CI
Age_at_diagnosis	0.008	1.03	[1.006–1.045]	0.309	1.01	[0.988–1.039]
Age ≥ 70 years	0.038	1.68	[1.030–2.753]			
NSAIDs_administration	0.003	0.47	[0.288–0.775]	0.023	0.43	[0.211–0.892]
Neoadj_chemotherapy	0.002	2.22	[1.336–3.689]	0.331	1.41	[0.704–2.833]
Preop_NLR > 3.53	0.143	1.63	[0.847–3.142]			
Residual_disease	0.016	2.38	[1.176–4.815]	0.386	1.76	[0.490–6.347]

**Table 3:** Postoperative complications.

		Patients with Ketorolac N = 112	Patients w/o Ketorolac N = 54	Total N = 166	*p*-Value
Postop complication	Missing	1 (0.9%)	0 (0.0%)	1 (0.6%)	0.169
	No	87 (77.7%)	27 (68.5)	124 (74.7%)	
	Yes	24 (21.4%)	17 (31.5%)	41 (24.7%)	

**Table 4:** Details of postoperative complications.

		Patients with Ketorolac N = 112	Patients w/o Ketorolac N = 54	Total N = 166	*p*-Value
Type of complications					
Urinary infection	No	20 (83.3%)	11 (64.7%)	31 (75.6%)	0.270
	Yes	4 (16.7%)	6 (35.3%)	10 (24.4%)	
Anaemia	No	18 (75.0%)	9 (52.9%)	27 (65.9%)	0.189
	Yes	6 (25.0%)	8 (47.1%)	14 (34.1%)	
Kidney failure	No	24 (100.0%)	16 (94.1%)	40 (97.6%)	0.415
	Yes	0 (0.0%)	1 (5.9%)	1 (2.4%)	
Veinous phlebitis (excluding superficiale phlebitis)	No	22 (91.7%)	16 (94.1%)	38 (92.7%)	0.999
	Yes	2 (8.3%)	1 (5.9%)	3 (7.3%)	
Pulmonary Thrombo-embolism	No	24 (100.0%)	16 (94.1%)	40 (97.6%)	0.415
	Yes	0 (0.0%)	1 (5.9%)	1 (2.4%)	
Secondary haemorrhage	No	24 (100.0%)	15 (88.2%)	39 (95.1%)	0.166
	Yes	0 (0.0%)	2 (11.8%)	2 (4.9%)	
Pneumothorax	No	17 (100.0%)	40 (97.6%)	23 (95.8%)	0.999
	Yes	1 (4.2%)	0 (0.0%)	1 (2.4%)	
Fecalis pertinonitis	No	19 (79.2%)	16 (94.1%)	35 (85.4%)	0.373
	Yes	5 (20.8%)	1 (5.9%)	6 (14.6%)	
Biliairis peritonis	No	24 (100.0%)	17 (100.0%)	41 (100.0%)	NA
Chylorrhea	No	22 (91.7%)	15 (88.2%)	37 (90.2%)	0.999
	Yes	2 (8.3%)	2 (11.8%)	4 (9.8%)	
Lymphorrhea	No	23 (95.8%)	15 (88.2%)	38 (92.7%)	0.560
	Yes	1 (4.2%)	2 (11.8%)	3 (7.3%)	
Duodenum leakage	No	24 (100.0%)	16 (94.1%)	40 (97.6%)	0.415
	Yes	0 (0.0%)	1 (5.9%)	1 (2.4%)	
Pancreatic fistula	No	23 (95.8%)	17 (100.0%)	40 (97.6%)	
	Yes	1 (4.2%)	0 (0.0%)	1 (2.4%)	0.999
Abdominal wall complication (evisceration, necrosis...)	No	22 (91.7%)	17 (100.0%)	39 (95.1%)	0.502
	Yes	2 (8.3%)	0 (0.0%)	2 (4.9%)	
Other	No Yes	17 (70.8%) 7 (29.2%)	8 (47.1%) 9 (52.9%)	25 (61.0%) 16 (39.0%)	0.195
Gastroparesis/Postop ileus	No	5 (20.8%)	5 (29.4%)	10 (24.4%)	0.633
	Yes	2 (8.3%)	4 (23.5%)	6 (14.6%)	
S. aureus sepsis	No	5 (20.8%)	9 (52.9%)	14 (34.1%)	0.175
	Yes	2 (8.3%)	0 (0.0%)	2 (4.9%)	
Cardiac arrhythmia	No	7 (29.2%)	8 (47.1%)	15 (36.6%)	0.999
	Yes	0 (0.0%)	1 (5.9%)	1 (2.4%)	
All other	No	4 (16.7%)	5 (29.4%)	9 (22.0%)	0.999
	Yes	3 (12.5%)	4 (23.5%)	7 (17.1%)	

**Table 5:** Dindo–Clavien classification.

		Patients with Ketorolac N = 24	Patients without Ketorolac N = 17	Total N = 41	*p*-Value
Dindo–Clavien classification	Missing	6 (25.0%)	6 (35.3%)	12 (29.3%)	0.815
	I	4 (16.7%)	4 (23.5%)	8 (19.5%)	
	II	7 (29.2%)	4 (23.5%)	11 (26.8%)	
	III	4 (16.7%)	2 (11.8%)	6 (14.6%)	
	IV	2 (8.3%)	0 (0.0%)	2 (4.9%)	
	V	1 (4.2%)	2 (4.9%)		

## Data Availability

All the data are kept by the first authors of the paper.
